# Direct Air Capture of Carbon Dioxide into MFI Frameworks by Low‐Temperature Swing Under Realistic Humidity

**DOI:** 10.1002/smll.202508150

**Published:** 2025-11-14

**Authors:** Sebastian Löbner, Ashour A. Ahmed, Majid Namayandeh Jorabchi, Alexander Wotzka, Marion Stöhr, Oliver Gröger, Christine Schütz, Marc Rüggeberg, Sebastian Wohlrab, Ali M. Abdel‐Mageed

**Affiliations:** ^1^ Leibniz‐Institute for Catalysis Albert‐Einstein‐Straße 29a 18059 Rostock Germany; ^2^ Group Innovation‐CO2 Economy Volkswagen AG 38436 Wolfsburg Germany

**Keywords:** adsorption, CO_2_‐direct‐air‐capture, molecular dynamics, Monte Carlo simulations, zeolites

## Abstract

The urgent need to combat global warming inspired the introduction of the concept of carbon dioxide direct air capture (CO_2_‐DAC), a key strategy for decentralized greenhouse gas removal from air. In this study, a simple approach for CO_2_‐DAC is introduced, utilizing ZSM‐5 and its ion‐exchanged analogues to effectively concentrate CO_2_ from humid air (80% RH) at 5 °C and enable its recovery at a moderate desorption temperature of 25 °C where in both cases, ambient air served as a sustainable CO_2_ source and desorption medium. Repeated adsorption (5 °C) and desorption (25 °C) cycles confirm the adsorbent stability under realistic conditions. Based on Monte Carlo (MC) and molecular dynamics (MD) simulations, a preferential CO_2_ adsorption via electrostatic interactions with minimal competitive adsorption with H_2_O is observed, explained by the facile diffusion of CO_2_ into the micropores compared to the preferred H_2_O adsorption on the outer surface of the zeolite. These experimental and theoretical insights pave the way for industrial CO_2_‐DAC applications using zeolite‐based adsorbents.

## Graphical abstract

Direct‐air‐capture of CO_2_ is a crucial strategy for mitigating global warming and ensuring a sustainable C‐supply to produce fuels and chemicals. This study explores MIF frameworks for CO_2_ capture from air under 80% RH and a low adsorption‐desorption temperature window, demonstrating high humidity tolerance and cyclic stability. Experiment and MC/MD simulations reveal preferential CO_2_ adsorption, facilitated by more facile CO_2_ diffusion into the MIF framework compared to water, preferentially adsorbed on the surface.

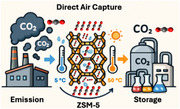

## 1 Introduction

The rapid progression of climate change, driven primarily by rising carbon dioxide emissions, coupled with the resulting socio‐economic problems, poses an unprecedented challenge to the sustainable development of the global economy, most notably for energy‐intensive heavy industries. According to the Paris Agreement, these effects are only avoidable in the foreseeable future if global warming can be strictly limited to well below 2 °C compared to the timeframe 1850 till 1900.^[^ ^1^, ^2^, ^3^ ^]^ The achievement of this goal can be assisted by the capture and recycling of the emitted carbon dioxide. By coupling CO_2_‐DAC technology with green hydrogen production, CO_2_ can be used as a building block for making platform chemicals (e.g., methanol or syngas) and consequently green fuels. This is anticipated to facilitate the transition from a fossil‐based economy to a cyclic carbon economy wherein CO_2_ is constantly reused together with green hydrogen, produced from wind and solar energies, for chemical production.^[^ ^4^, ^5^, ^6^ ^]^ It is thus imperative to capture tremendous amounts of CO_2_ from the air to reach both the climate and industry goals.^[^ ^3^, ^7^ ^]^ The first step for achieving this integral target is the development of sustainable and suppliable materials and processes to efficiently implement decentralized CO_2_ capture from air or from the outlet flue gas of industrial emissions.^[^ ^8^ ^]^

The CO_2_‐DAC technology is based on the adsorption of small CO_2_ concentrations (≈400 ppm) from ambient air in the presence of water vapour (i.e., humidity) and other interfering atmospheric constituents.^[^ ^1^, ^2^ ^]^ Therefore, the challenges of this process can be articulated as: i) the competitive adsorption of water with CO_2_ on active adsorption materials, and ii) the energy input needed to cool the adsorption unit (mostly at temperatures ≤ 0 °C) or for the thermal desorption of CO_2,_ which is typically done at relatively high temperatures (>100 °C). The commonly studied adsorbent materials rely on medium to strong chemisorption of CO_2_ on alkali metal carbonates, oxides, or amines.^[^ ^9^, ^10^, ^11^ ^]^ Despite achieving a higher adsorption capacity than other solid adsorbents such as zeolites, these materials require higher energy input in the thermal desorption step to recover CO_2_. In contrast, physisorption‐based adsorbents can achieve desorption at much lower temperatures (e.g., zeolites and carbon‐based materials).^[^ ^10^ ^]^ This leads to a narrow “adsorption‐desorption” temperature window, helping to minimize the energy input in the DAC process (see concept illustrated in Figure S1, Supporting Information). The main challenge in using these materials is the need to perform the adsorption step at relatively low temperatures (below 0 °C), and at the same time, these adsorbents often have a lower adsorption capacity compared to the basic chemisorbents mentioned above.

Zeolites are typically used in high‐pressure/temperature flow reactors for the capture of high concentrations of CO_2_^[^ ^12^ ^]^ or as CO_2_ separation membranes.^[^ ^13^ ^]^ For CO_2_‐DAC, study and understanding of their adsorption and desorption characteristics, as well as their molecular interactions with CO_2_ and H_2_O under CO_2_‐DAC conditions, is therefore essential to advance them toward potential technical applications.

In this contribution, we show for the first time that the MFI zeolite (ZSM‐5) and its ion‐exchanged forms (Zn, Mg, and Ca) can be selectively used, under cyclic operations, for CO_2_‐DAC at ambient pressure and a temperature of 5 °C in the presence of 80% relative humidity (RH). In this process, air can additionally be used to release the adsorbed CO_2_ at a rather low desorption temperature (25 °C), eliminating the need for expensive inert gases (Ar or N_2_) typically used for this purpose, which reduces the energy cost for the whole CO_2_‐DAC process. This concept, characteristic of MIF zeolites, has been recently patented for potential technical applications (see ref. [14]). These insights were supported and further explained by MC and MD simulations, which helped to clarify the competitive adsorption behavior of CO_2_ with H_2_O and its diffusion into micropores.

## 2 Results and Discussion

We employed a commercially available ZSM‐5 zeolite after modifications (for all syntheses and adsorption experiments, see Methods, Supporting Information; ref. [15]). The pristine ZSM‐5 framework, purchased in its NH_4_ form (NH_4_‐ZSM‐5; Si/Al = 15), was modified additionally through ion‐exchange with Ca^2+^, Mg^2+^, and Zn^2+^ cations (see Table S1, Supporting Information). Next, we examined the porosity of the four samples (ZSM‐5, Ca‐ZSM‐5, Mg‐ZSM‐5, Zn‐ZSM‐5) using N_2_ adsorption experiments. All samples showed a Type I adsorption isotherm according to IUPAC nomenclature (see Figure S2, Supporting Information). Based on the BET model, we calculated small differences in the specific surface areas (SSA), ranging from 390 m^2^ g^−1^ for Zn‐ZSM‐5 to 407 m^2^ g^−1^ for Mg‐ZSM‐5 and ZSM‐5 (see Table S1, Supporting Information). The plot of pore size distribution with respect to the incremental SSA showed that the average pore diameter is ≈0.84 nm (see details in Table S1, Supporting Information).

Next, we investigated the thermal behavior of all zeolite samples using thermogravimetric analysis (TGA). All fresh samples, without any prior treatment, exhibited two distinct temperature steps (see Figure S3a, Supporting Information). The first step was completed within 40 min at a temperature of 220 °C, during which a mass loss ranging from 4.6% (Zn) to 7.2% (NH_4_) was recorded. In the second step, between 220 and 500 °C, a significantly smaller mass loss was observed, ranging from 0.7% (Zn‐ZSM‐5) to 1.3% (Mg‐ZSM‐5). After prolonged exposure to 500 °C, the mass remained constant for Ca‐ZSM‐5 and Mg‐ZSM‐5. However, NH_4_‐ZSM‐5 and Zn‐ZSM‐5 both exhibited an additional mass loss of roughly 0.7%. This final mass loss could be attributed to the slow de‐ammoniation of strong acid sites to form H‐ZSM‐5 or the evaporation of zinc (vapour pressure = 0.0137 mmHg at 354 °C^[^ ^16^ ^]^), respectively. Afterwards, we cooled the sample down to room temperature under nitrogen and then exposed it to ambient air for 2 h to examine the reversibility of the mass gain, presumably from humidity and CO_2_ capture. Upon repeating the TGA experiment, we observed a similar mass loss across all ZSM‐5 adsorbents (see Figure S3b, Supporting Information), although there were slight differences in the rate of mass loss with temperature. As a blind test, we maintained the Mg‐ZSM‐5 sample under a flow of N_2_ for 16 h within the device at room temperature after the second measurement before repeating the TGA experiment (see Figure S4, Supporting Information). This time, the zeolite showed no significant mass loss. This indicates that the observed mass loss after air exposure resulted from the adsorption of CO_2_ and H_2_O from air. To discriminate between the two species, an additional experiment was performed. After the desorption of species from ambient air, the sample was exposed to a stream of moisture‐free 400 ppm CO_2_ in N_2_ at 25 °C (see Figure S5a, Supporting Information). During the latter step, no significant mass change occurred (see Figure S5, Supporting Information). Based on these experiments, we conclude that the major mass loss is due to H_2_O and that desorption occurs below the temperature of ≈150 °C. Therefore, a drying step before the dynamic CO_2_ adsorption experiments should be conducted at a minimum temperature of 150 °C.

In the dynamic CO_2_ adsorption experiments, CO_2_ capture is done under a flow of ambient air with a realistic humidity level (80%), following a breakthrough measurement.^[^ ^14^, ^17^, ^18^ ^]^ The experiment starts with an adsorption step at 5 °C by exposing the adsorbent bed packed in a flow reactor to a continuous flow of synthetic air (0.04% CO_2_, 21% O_2_, 78.96% N_2_) in the absence of H_2_O vapour. When water is present, the other gas components change accordingly (RH of 80% at 5 °C is equivalent to 0.662 kPa H_2_O). In the beginning, CO_2_ is completely adsorbed (see Figure S1b, Supporting Information). The end of the adsorption step is marked by a breakthrough in the curve. The air flow containing CO_2_ is continued beyond the initial breakthrough point to reach saturation at a CO_2_ concentration of 400 ppm (0.04%). An isothermal desorption step is then carried out at 25 °C under continuous Ar flow, where CO_2_ desorbing from the adsorbent bed is monitored over time until no further CO_2_ is detected. In our experiments, we could replace Ar by air. The effluent gases are continuously monitored using an online mass spectrometer connected to the outlet of the CO_2_‐capture reactor.

**Figure 1:**
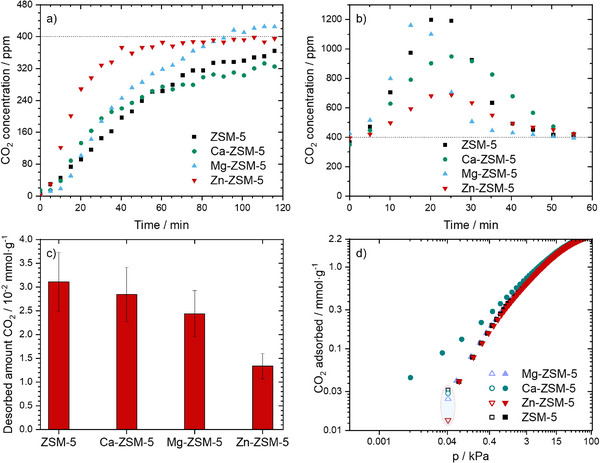
a) Comparison of the breakthrough adsorption curves of different adsorbents (5 g) at 5 °C using 200 mL min^−1^ synthetic air (0.04% CO_2_, 21% O_2_, 78.96% N_2_). b) Desorption of CO_2_ after the capture step under a flow of Ar (100 mL min^−1^) at ambient pressure and 100 °C. c) Amount of CO_2_ desorbed from different adsorbents. d) Plot of the amount of CO_2_ adsorbed during static (manometric) CO_2_ adsorption measurements (block symbols) and the value obtained from CO_2_ desorption under dynamic (flow) conditions (hollow symbols).

In the dynamic CO_2_ adsorption experiments, we first performed the capture of CO_2_ in a dry synthetic air gas mixture (0% RH). The comparison of the adsorption part and breakthrough edge indicated only a limited effect of the ion exchange on the adsorption properties of ZSM‐5 (**Figure** **1**). As shown there, the adsorption step is nearly complete after ≈80 min for the Zn‐ and Mg‐ZSM‐5. At extended exposure times, no further change in CO_2_ levels was observed for these two samples (Figure 1a). Ca‐ZSM‐5 and ZSM‐5 were continuously increasing over the whole 120 adsorption time. The CO_2_ concentration of the gas stream reached ≈320 and 360 ppm, respectively. Next, we tried to use only dry air to release the adsorbed CO_2_. We performed isothermal desorption by heating the reactor to 100 °C under a flow of air (i.e., in the presence of CO_2_) at ambient pressure.

This approach (i.e., using dry air for desorption step) reduces the running cost of any potential CO_2_‐DAC application by eliminating the need for pure gases such as Ar, He, or N_2_. As shown in Figure 1b, the CO_2_ concentration increases with time during the desorption step, starting at ≈400 ppm (0 min) and reaching a maximum of ≈700–1200 ppm at ≈ 20 min. At extended desorption time, the CO_2_ concentration decreases and returns to 400 ppm after 58 min on stream. Quantifying the adsorbed CO_2_ amount based on the corresponding MS signal during the runtime (Figure 1c; Table S2, Supporting Information), indicated that the amount of CO_2_ collected on different materials follows the order: ZSM‐5 > Ca‐ZSM‐5 > Mg‐ZSM‐5 > Zn‐ZSM‐5. Notably, the Ca‐ZSM‐5 (28 µmol g^−1^) and Mg‐ZSM‐5 (24 µmol g^−1^) adsorbent samples showed only slightly reduced adsorption capacity compared to the pristine ZSM‐5 (31 µmol g^−1^). In contrast, Zn‐ZSM‐5 exhibited a much lower adsorption capacity (14 µmol g^−1^), suggesting that the exchange of the ions inside the ZSM‐5 framework has a negative effect on their adsorption capacity for CO_2_.

Additionally, we compared the amount of CO_2_ captured under dynamic conditions to the amount adsorbed during static isothermal CO_2_ adsorption measurements to assess possible differences between both measurement regimes.^[^ ^17^ ^]^ The CO_2_ adsorption isotherms were measured at 0 °C (see Figure 1d). The comparison of both experiments shows that static experiments result in slightly higher CO_2_ uptake, particularly for Ca‐ZSM‐5, which has slightly smaller pores as indicated by the lower equilibrium pressure for similar amounts adsorbed below ≈3 kPa. In general, both methods yield adsorption values of the same magnitude at 0.04 kPa. This suggests that dynamic adsorption experiments remain a reliable method for evaluating CO_2_ capture on these materials.

**Figure 2:**
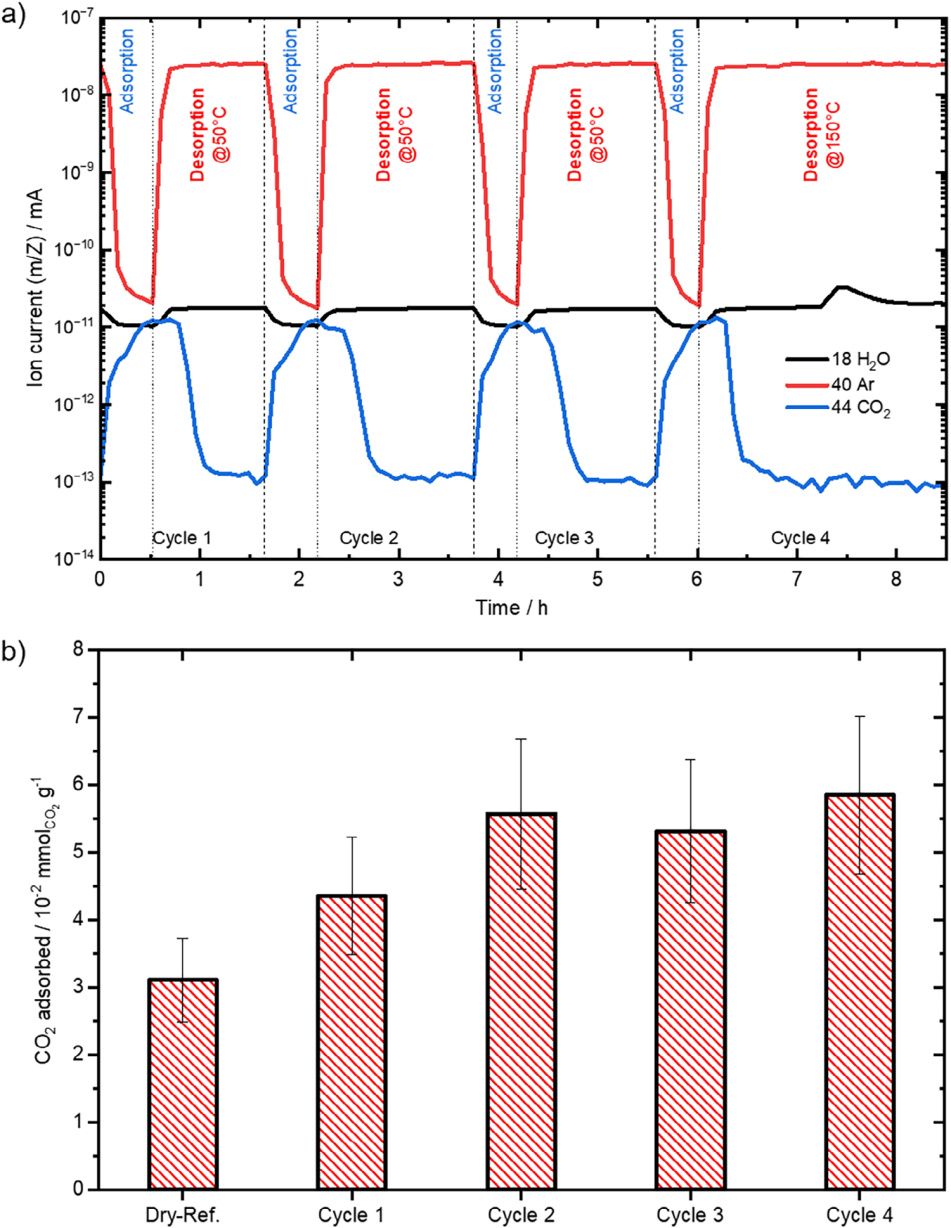
Repeated dynamic adsorption/desorption cycles on ZSM‐5 (0.5 g), including the sequential adsorption at 5 °C from humidified air (Air with a RH of 80% ≈ 0.662% H_2_O at 5 °C; flow rate: 100 mL min^−1^) followed by the desorption in Ar at 25 °C; during cycle 4, the desorption temperature was 150 °C. (a: plot of ion current during the dynamic swing experiments; b: the quantified amount of CO_2_ adsorbed during each cycle (based on desorption) together with the measurement with dry air shown for comparison (Dry‐Ref.).

Next, we examined the impact of humidity on the adsorption capacity of ZSM‐5 while also assessing its cyclic stability; both aspects are essential for industrial applications. The adsorbent was subjected to four consecutive adsorption (5 °C)/desorption (25 °C) cycles. We observed that the CO_2_ adsorption breakthrough curve repeated at 25 min, with complete desorption occurring within 30 min (**Figure** **2**a). The highly reversible adsorption/desorption cycles indicate the efficiency of this process within the tested temperature range (5–25 °C). By quantifying the adsorption capacity over the four cycles (5.3 ± 0.6 10^−2^ mmol g^−1^ on average) and comparing it to that under dry conditions (3.1 10^−2^ mmol g^−1^), we found that the presence of humidity increases the adsorption performance (Figure 2b). However, the amount of water detected with the MS did not change significantly within each step. Only during the last desorption step did water desorb at higher temperatures, as indicated by the peak ≈7.5 h. With respect to the duration of each cycle, we calculated a productivity of ≈30.2 g_CO2_∙kg^−1^∙d^−1^ (0.69 mol_CO2_∙kg^−1^∙d^−1^). These findings demonstrated the excellent humidity tolerance of ZSM‐5 and its strong potential for CO_2_‐DAC under cyclic operation conditions.

### 2.1 Computational Results

**Figure 3:**
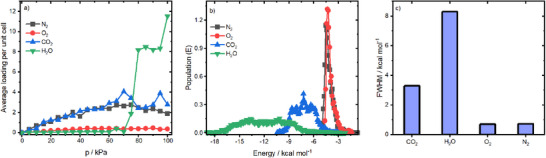
a) Simulated adsorption isotherms of a gas mixture containing 72.48% N_2_, 13.52% O_2_, 4% CO_2_, and 10% H_2_O in the modified MFI (ZSM‐5) structure at 5 °C. b) Energy distribution of the adsorption of different gas molecules into the MFI framework at 5 °C. c) Full width at half maximum (FWHM) for different peaks plotted in layer (b).

To better understand the mutual effects of co‐adsorbing molecules in realistic synthetic air composition, particularly the H_2_O‐CO_2_ co‐adsorption, which is not fully accessible through experiments, we conducted computations supporting the experimental findings. First, we simulated the adsorption of components from a gas mixture consisting of 72.48% N_2_, 13.52% O_2_, 4% CO_2_, and 10% H_2_O across a pressure range of up to 100 kPa (see Methods, Supporting Information; ref. [19]). We increased the CO_2_ concentration to 4% on purpose so that the simulation is sensitive enough to detect potential co‐adsorption effects. The isotherms in **Figure** **3**a show a rapid increase in CO_2_ uptake at low pressures, followed by a plateau as pressure rises further, indicating the saturation of adsorption sites, which is typical for microporous materials. In contrast, despite their higher concentration and stronger interactions with the zeolite, water molecules showed limited adsorption below 70 kPa. Above 70 kPa, the average loading increased steadily with pressure, rising by more than an order of magnitude at 100 kPa. This observation can be explained by the clustering of water in the micropores, particularly at higher pressures,^[^ ^20^ ^]^ as in our adsorption experiment. Interestingly, this did not have an apparent effect on CO_2_ adsorption. This is in accordance with previous literature reports, as both molecules adsorb on different sites. While water adsorbs primarily at the Lewis‐ and Brønsted‐acidic sites,^[^ ^21^ ^]^ CO_2_ adsorbs at the zeolite wall or (if present) metal counterions.^[^ ^22^ ^]^ The O_2_ and N_2_ simulated isotherms are flatter than CO_2_ and H_2_O, indicating more limited interaction with the MFI structure, which is expected for nonpolar gases adsorbing on microporous materials.^[^ ^23^ ^]^ The adsorption isotherms were also studied at 25 and 50 °C. At these higher temperatures, less CO_2_ was adsorbed, consistent with experimental data and literature. At 5 °C, the loading increases with pressure (see Figures S6 and S7, Supporting Information). Unlike the adsorption at 5 °C, water adsorption is much more limited at 25 and 50°C. This agrees with the limited effect of H_2_O on the adsorption capacity of CO_2_ in the metal‐loaded ZSM‐5 frameworks at these temperatures, in agreement with our experiments.

For deeper insight from these simulations, we plotted the energy distribution of the adsorption energies for CO_2_, O_2_, N_2_, and H_2_O in the MFI structure (see Figure 3b). For CO_2_, the peak maximum occurs at a less negative energy (−7.25 kcal mol^−1^), compared to water (−13.5 kcal mol^−1^) hinting at stronger H_2_O adsorption than CO_2_, whereas both gases adsorb stronger than O_2_ and N_2_. Additionally, water shows two maxima. The two peaks in the curve for H_2_O likely arise from the presence of distinctly different adsorption sites within the MFI zeolite framework. MFI zeolites, such as ZSM‐5, typically contain a combination of hydrophilic sites (e.g., Brønsted acid sites associated with Al substitutions) and more hydrophobic regions in the siliceous channels and intersections. Water molecules bind more strongly (more negative energies, corresponding to the left peak ≈−16 to −12 kcal mol^−1^) at the hydrophilic sites, often forming small clusters like dimers at low coverages. In contrast, weaker binding (less negative energies, the right peak ≈−12–−8 kcal mol^−1^) would occur in the hydrophobic pore interiors or as larger clusters form at higher loadings. This heterogeneity in site energies leads to a bimodal distribution, unlike the more uniform physisorption seen for non‐polar gases like N_2_ and O_2_. Monte Carlo simulations of water adsorption in MFI support this, showing high heats of adsorption at low coverage due to preferential coordination at hydrophilic sites.^[^ ^24^ ^]^ This conclusion agrees with MC simulations discussed later.

The full width at half maximum (FWHM) for CO_2_ is significantly smaller (3.3 kcal mol^−1^) compared to water (8.3 kcal mol^−1^) (see Figure 3c). This suggests that CO_2_ binds to more specific sites, whereas water adsorption spreads across a larger number of sites. This proposal aligns well with the findings by Wirawan et al., reporting a single type of interaction of CO_2_ with ZSM‐5 frameworks.^[^ ^25^ ^]^ The overlap between both peaks, CO_2_ and water, occurs at energies between −10.35 and −6.65 kcal mol^−1^, which is largely below the CO_2_ peak maximum (−7.25 kcal mol^−1^), indicating limited competitive adsorption with water. The energy distributions for O_2_ and N_2_ are narrower and shifted toward 5 kcal mol^−1^, reflecting much weaker interactions with the MFI structure compared to CO_2_ and H_2_O. This aligns with the non‐polar nature of O_2_ and N_2_, making their adsorption less favourable than CO_2_ and H_2_O. Overall, the simulations show a more selective CO_2_ adsorption into the MFI framework with minimal interference from H_2_O, confirming that CO_2_ binds to specific sites with limited competitive adsorption from H_2_O.

Although experiments and Monte Carlo simulations show distinct CO_2_ uptake and H_2_O co‐adsorption behavior in MFI‐based materials, the molecular mechanisms remain unclear. The question remains why CO_2_ adsorbs preferentially in the presence of moisture under ambient conditions. We performed nine MD simulations on three systems: 60 CO_2_, 60 H_2_O, and a 1:1 mix of 30 CO_2_ and 30 H_2_O molecules. Each system was started on three MFI zeolite surface planes ([100], [001], [010]) to examine surface effects. (see Methods, Figure S8 (Supporting Information) and ref. [26, 27]). These simulations reveal how surface orientation, electrostatics, and dispersion forces jointly affect CO_2_ adsorption, H_2_O–CO_2_ co‐adsorption, and directional transport within the zeolite.

**Figure 4:**
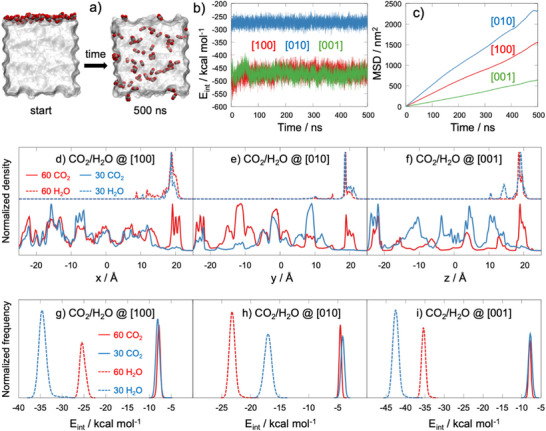
a) Side‐view snapshots (along [010]) of 60 CO_2_ molecules initially adsorbed on the [100] surface at simulation start (left) and end (right). MFI surface: transparent; CO_2_: gray C, red O. b) Total interaction energy of 60 CO_2_ molecules with MFI surfaces along [100], [010], and [001]. c) Mean square displacement (MSD) of CO_2_ within surface planes. d–f) Normalized partial density profiles for CO_2_ and H_2_O across [100], [010], and [001] surfaces. Origin (0 Å) = surface plane center. g–i) Interaction energy distributions per molecule for CO_2_ (solid) and H_2_O (dashed) with MFI surfaces. Panels (d–i) summarize nine MD simulations: Pure 60 CO_2_, Pure 60 H_2_O, and 30 CO_2_ + 30 H_2_O mixture on [100], [010], and [001] surfaces.

For the system containing 60 CO_2_ molecules, CO_2_ is adsorbed initially on the MFI surface (e.g., see adsorption on the [100] plane in **Figure** **4**a; Figure S9, Supporting Information). Across all surface planes, CO_2_ molecules spilled over from surface sites into the zeolite micropores, redistributing throughout the MFI framework. This behavior is evident in the partial density profiles, which reflect the spatial distribution of CO_2_ along the x‐, y‐, and z‐axes of the framework (Figure 4d–f; additional details in Figure S10, Supporting Information). Among the three surfaces, the [001] plane exhibited notably higher CO_2_ density ≈±20 Å along the z‐axis (Figure 4f), indicating stronger physisorption affinity (Figure 4b). This observation correlates well with the lower CO_2_ mobility along the [001] direction as seen in the mean square displacement (MSD) data (Figure 4c).

In terms of interaction strength, the average interaction energy per CO_2_ molecule with the MFI surfaces followed the order: [010] (−4.6 kcal mol^−1^) < [100] ≈ [001] (−7.9 kcal mol^−1^) (Figure 4b). Electrostatic interactions primarily drive CO_2_ binding along the [100] and [001] planes, contributing −5.3 and −5.7 kcal mol^−1^, respectively. In contrast, dispersion forces dominate the interaction along the [010] surface, accounting for −3.4 kcal mol^−1^. This weaker total binding energy and lower Coulomb interactions, coupled with a dominance of dispersion interactions, can be attributed to the larger pore size of the [010] surface compared to the [100] and [001] surfaces (see Figure S8, Supporting Information). These structural characteristics and the weaker binding facilitate enhanced CO_2_ mobility within the MFI, as evidenced by the higher mean square displacement (MSD) values observed along the [010] surface (Figure 4c). In contrast, the [001] surface exhibited the lowest diffusion rates due to its stronger binding affinity, while the [100] surface demonstrated intermediate behavior. Additional insights into the MSD components along each dimension (x, y, and z) and across planes (xy, xz, and yz) for CO_2_ on the three MFI surface planes are provided in Figure S11 (Supporting Information). Overall, these findings underscore that CO_2_ uptake and mobility in MFI zeolites are dictated by surface‐dependent physisorption mechanisms, where the relative contributions of electrostatic and dispersion interactions play a decisive role.

A detailed analysis of CO_2_’s orientation relative to the MFI framework reveals that its O atoms consistently lie closer to the framework's Si and O atoms than its C atom. This is confirmed by radial distribution functions for O(CO_2_)–Si(MFI), C(CO_2_)–Si(MFI), O(CO_2_)–O(MFI), and C(CO_2_)–O(MFI) pairs (Figure S12, Supporting Information). Complementary charge density profiles along the x‐, y‐, and z‐axes of the three MFI surface planes further show that the negatively charged oxygen atoms approach the framework atoms more closely than the positively charged carbon (Figures S13–S15, Supporting Information). Together, these findings highlight the pivotal role of CO_2_’s O atoms in interacting with the MFI framework, shedding light on the underlying adsorption mechanism.

Analysis of simulations for both pure H_2_O (60 molecules) and a 1:1 CO_2_/H_2_O mixture (30 molecules each) revealed that water adsorption does not significantly influence CO_2_ capture or diffusion along MFI framework directions (Figure 4d–i and Figure S16, Supporting Informations). Unlike CO_2_, H_2_O molecules remained predominantly adsorbed near the MFI surface (≈20 Å) in both pure and mixed systems and across all surface planes, exhibiting minimal diffusion, i.e., low H_2_O mobility into the micropores (Figure 4d–f). This strong localization arises from H_2_O's higher binding affinity to MFI surfaces (−17.0–−42.5 kcal mol^−1^) compared to CO_2_, evidenced by the broader, surface‐dependent distribution of interaction energies per single H_2_O molecule (Figure 4g–i), which agrees well MC simulation results. Critically, while varying H_2_O concentration significantly alters its own interaction energy with the surface, it has no measurable effect on CO_2_ adsorption or CO_2_‐surface interaction energy (Figure 4g–i) under the simulation conditions. Moreover, the wider FWHM of H_2_O's interaction energy distribution, compared to CO_2_’s sharper distribution, highlights H_2_O's surface‐dependent binding heterogeneity.

Additionally, based on our surface‐resolved MD across the three crystallographic planes, we find that, even for the same MFI model (identical atoms/coordinates) and the same composition (30 CO_2_ + 30 H_2_O, Figure S17, Supporting Information), the H_2_O adsorption‐energy distribution clearly shows three well‐defined peaks (see Figure 4g–i). This reflects strong plane dependence of H_2_O binding: the [100], [010], and [001] surfaces each produce a distinct H_2_O interaction‐energy subpopulation, which is consistent with different hydrogen‐bonding motifs and local environments. While absolute energies from MD and MC differ (as expected from methodological/ensemble differences), the qualitative multi‐peak structure is consistent. Moreover, Figure S17 (Supporting Information) maps the hydrogen‐bond networks and their distributions on [100]/[010]/[001], corroborating the plane‐dependent H_2_O binding that gives rise to the observed multi‐peak behavior. The interactions of adsorbed water molecules (e.g., clustering within micropores) are also very likely to depend strongly on adsorption temperature, which remains an open question for future investigation.

To sustain our insights, we calculated the radial distribution function (RDF) of adsorbates relative to the MIF framework to locate the relative population of both CO_2_ and H_2_O on the active adsorption sites. In Na‐form MFI, the direction‐resolved RDFs, g(r), reveal that along the [100] and [010] planes, the first‐ coordination shell of water oxygens (Ow) acts primarly to framework Si and Al ions into the MIF structure, with Si dominating in the [100] plane and Al in the [010] plane, followed by a stronger Na⁺ first‐shell peak and weaker Ow–OMFI (OMFI: framework O sites) interactions, see Figure S18,b, Supporting information. In contrast, along the [001] plane, the earliest and most pronounced feature is Ow–Si, then Ow–OMFI (see Figure S18a–c, Supporting Information). For water hydrogens (Hw), the closest approach in all three directions is to OMFI (low‐intensity hydrogen‐bond peaks), followed by a strong Hw–Na⁺ peak in [100]/[010] and Hw–Si in [001] (see Figure S18d–f, Supporting Information). CO_2_ exhibits similar behavior across [100], [010], and [001], with its primary first shell defined by OCO_2_–Na⁺ (and CCO2–Na⁺ at longer distance (r)), while OCO_2_–Si/Al features are secondary and substantially weaker (see Figure S18g–l, Supporting Information). These trends establish that metal cations act as the dominant adsorption sites, whereas Brønsted acid sites (H+) are effectively absent in the Na‐exchanged material and thus negligibly occupied. Examining the RDFs for Na+ into the MIF framework, we also noted that the Na+ reside more preferentially on the Al sites rather than Si, as indicated by the higher intensity of the Na‐Al shell in the radial distribution plots (see Figure S19, Supporting Information). Finally, residence time analysis shows that H_2_O generally exhibits longer and more stable adsorption at hydrogen‐bond and van der Waals sites compared to the shorter and more transient adsorption of CO_2_ within the MIF micropores (see detailed description of the occupancy and residence‐time analysis in the Supporting Information).

In total, these findings from MD simulations demonstrate that the stronger, surface‐dependent binding of H_2_O reduces its mobility, whereas the mobility of CO_2_ within the MFI framework remains largely unaffected. This mechanistic interpretation is consistent with current experimental observations and Monte Carlo simulation results, jointly providing a coherent explanation for the origin of CO_2_ adsorption selectivity in the presence of competing H_2_O under ambient conditions with 80% RH.

## 3 Conclusion

In this study, we evaluated ZSM‐5 and its ion‐exchanged forms (Zn^2+^, Mg^2+^, Ca^2+^) for CO_2_ capture from synthetic air containing 400 ppm CO_2_ and 80% relative humidity. The materials showed reversible CO_2_ physisorption at low temperatures and efficient desorption at 25 °C, reducing energy consumption encountered in high temperature processes. Under these realistic conditions, adsorption capacities varied by ≈33% among the adsorbents. During cyclic adsorption–desorption at 80% RH, ZSM‐5 displayed stable and reversible CO_2_ uptake/release. Molecular dynamics (MD) and Monte Carlo (MC) simulations provided further insight into co‐adsorption behavior of CO_2_ and H_2_O. MD simulations revealed dominant Coulombic interactions between CO_2_ and the MFI framework, while MC simulations showed pressure‐dependent competitive adsorption, favouring CO_2_ at lower pressures, consistent with experimental results showing minimal water interference. Insights from MD indicated also that CO_2_’s faster diffusion into micropores accounts for the observed CO_2_ selectivity, when compared to water's preferential outer‐surface adsorption. These findings support the use of these materials as cost‐effective and energy‐efficient options for CO_2_‐DAC applications under humid conditions.

## Conflict of Interest

The authors declare no conflict of interest.

## Supporting information



Supporting Information

## Data Availability

The data that support the findings of this study are available from the corresponding author upon reasonable request.
