# Supplementary material for: Direct Air Capture of Carbon Dioxide into MFI Frameworks by Low‐Temperature Swing Under Realistic Humidity
Source: Small. 2025 Nov 14;21(51):e08150. doi: 10.1002/smll.202508150 (PMC12723347; doi:10.1002/smll.202508150)
Supplement: Supplementary file 1 — Supporting Information [file SMLL-21-e08150-s001.pdf]

NANO · MICRO
small

Supporting Information

for *Small*, DOI 10.1002/smll.202508150

Direct Air Capture of Carbon Dioxide into MFI Frameworks by Low-Temperature Swing Under Realistic Humidity

*Sebastian Löbner, Ashour A. Ahmed, Majid Namayandeh Jorabchi, Alexander Wotzka, Marion Stöhr, Oliver Gröger, Christine Schütz, Marc Rüggeberg, Sebastian Wohlrab and Ali M. Abdel-Mageed**

Supplementary Information

Direct Air Capture of Carbon Dioxide into MFI Frameworks by Low-Temperature Swing under Realistic Humidity

Sebastian Löbner[a], Ashour A. Ahmed[a], Majid Namayandeh Jorabchi[a], Alexander Wotzka[a], Marion Stöhr[a], Oliver Gröger[b], Christine Schütz[b], Marc Rüggeberg[b],

Sebastian Wohlrab[a], Ali M. Abdel-Mageed[a*]

[a]*Leibniz Institute for Catalysis (LIKAT), Albert-Einstein-Str. 29a, 18059 Rostock, Germany*

[b]*Volkswagen Aktiengesellschaft, Group Innovation-CO_2 Economy, 38436 Wolfsburg, Germany*

Sections:

1.**Methods** S3
1.1 Experimental methods S3
1.1.1 Material synthesis: S3
1.1.2 Characterization of ion-exchanged ZSM-5 S3
1.1.3 Setup and procedure for the direct air capture (DAC) S4
1.2 Computational methods S4
1.2.1 Monte Carlo (GCMC) simulations S4
1.2.2 Molecular modeling and computational details S5

2. **Supporting Tables** S7
Table S1: Summary of textural properties and porosity of ion-exchanged M-ZSM-5 samples and the pristine material with a nominal molar ratio of Si/Al = 13.6. 9
Table S2: The amount of adsorbed CO_2 on different ZSM-5-based samples during the dynamic adsorption-desorption experiments. 9

3. Supporting Figures S8
Figure S1: (a) Illustration of the energy demand of the adsorption/desorption window in the CO_2-DAC process. (b) illustration of the breakthrough curve during the adsorption/desorption process in the capture of CO_2 under flow conditions. S10
Figure S2: N_2 adsorption/desorption isotherms of the as-prepared frameworks (a: H-ZSM-5; b: Mg-ZSM-5; c: Zn-ZSM-5; d: Ca-ZSM-5). Before each adsorption experiment, the sample was degassed for 2 h at 200 °C and 4 µbar. S11
Figure S3: (a) Thermogravimetric analysis (TGA; Left Y-axis: residual mass; right Y-axis: temperature; X-axis: time) of the as-prepared ZSM-5 adsorbents in N_2. (b) TGA profile of adsorbents after the first TGA profile (shown in part a) followed by cooling to RT in N_2 for 2 hours and subsequent exposure to ambient air for 2 h. S12
Figure S4: Thermogravimetric analysis (TGA) profile collected on the Mg-ZSM-5 adsorbent after a preceding TGA step followed by keeping the adsorbent under a flow of N_2 for 16 h at RT.....S13
Figure SS5: TG curves of Ca-ZSM-5 under a) pure nitrogen to desorb species from ambient air and b) N_2 (segment 1) and 400 ppm CO_2 in N_2 (segment 2). Dotted vertical lines indicate segment transistion. Between the measurements the sample was cooled to RT under N_2 in the device. S14
Figure S6: Simulated adsorption isotherms of the gas mixture (72.48 % N_2, 13.52 % O_2, 4 % CO_2, and 10 % H_2O) in the modified MFI structure at 25 °C. S15
Figure S7: Simulated adsorption isotherms of the gas mixture (72.48 % N_2, 13.52 % O_2, 4 % CO_2, and 10 % H_2O) in the modified MFI structure at 50 °C. S16
Figure S8: (a–c) Top views of the three modeled MFI surface planes, (d) schematic representation of the molecular modeling approach for CO_2 adsorption on the MFI surface, and (e) side view and (f) top view of the initial configuration with 60 CO_2 molecules 17
Figure S9: Side-view snapshots captured during the MD simulation of CO_2 adsorption on the [100] MFI surface plane 18
Figure S10: The partial density of CO_2 molecules along each dimension (x, y, and z) for the three MFI surface planes S19
Figure S11: The overall mean square displacement (MSD) in the xyz directions, along with its individual components in each single dimension (x, y, and z), and for plane (xy, xz, and yz) of CO_2 for the three MFI surface planes S20

Figure S12: The radial distribution function, g(r), of the distance between both C and O atoms of CO_2 and the MFI-O atoms (top three panels) and Si atoms (bottom three panels) S21
Figure S13: The charge density of the MFI (top three panels) and CO_2 (bottom three panels) along the axis perpendicular to the MFI surface plane is given in the corresponding panel. The center of the MFI surface was taken as zero along each respective axis. S22
Figure 14: The normalized charge density of both MFI and CO_2, as presented in Figure S12, along the axes perpendicular to the MFI surface planes given in the respective panel. S23
Figure S15: The charge density of CO_2 along the x-, y-, and z-axes for the three MFI surface planes. The center of the MFI surface was taken as zero along each respective axis. S24
Figure S16: Side-view snapshots from an MD trajectory showing co-adsorption of 30 CO_2 and 30 H_2O on the MFI [100] surface over time. Two visualization schemes help track uptake S25
Figure S17: Hydrogen bonding in CO_2/H_2O mixtures on MFI surfaces S26
Figure S18. Radial distribution functions, g(r), in ZSM-5 (MFI) for adsorbates relative to Na^+ and framework atoms (Si, Al, O), resolved by crystallographic direction S27
Figure S19. Radial distribution functions, g(r), for Na^+–framework pairs (Na–Si, Na–Al, Na–O) in ZSM-5 (MFI), resolved by crystallographic direction: (a) [100], (b) [010], (c) [001] S28
Additional explanation related to calculations in Figures S18/S19 S28
References S30

1. Methods:

1.1 Experimental methods

1.1.1 Material synthesis:

We used a ZSM-5 framework in its ammonium form purchased from Zeolyst International (batch no.: CBV3024E). This zeolite has a nominal Si/Al ratio of 15 according to the supplier (13.6 according to ICP-OES). Ion-exchanged zeolite (Zn^{2+}, Mg^{2+}, and Ca^{2+}) frameworks were prepared using chloride salts according to the following recipe. First, we sonicated 3 g of the ZSM-5 zeolite powder in 300 ml of deionized water for 30 min and heated the suspension to a temperature of 80 °C. Next, an amount of the chloride salt equivalent to a nominal loading of 1 wt.% (X) of different metals was dissolved in 50 ml of deionized water (X = 0.063 g $ZnCl_2$; 0.0829 g $CaCl_2$; 0.118 g $MgCl_2$). After that, we added the metal salt solution dropwise to the zeolite suspension under continuous stirring at room temperature. The mixture was then heated to 80 °C and stirred for 24 h. Next, the M-ZSM-5 was filtered out and washed five times with deionized water. Finally, the obtained solid was dried under a vacuum overnight.

1.1.2 Characterization of ion-exchanged ZSM-5:

The actual metal loading of the metal ions was determined by inductively coupled plasma optical emission spectroscopy (ICP-OES) measurements using a 715-ES ICP ES (Varian, Palo Alto, CA). For these experiments, about 10 mg of a sample were dispersed in a mixture of aqua regia and HF and subsequently digested in the microwave-assisted sample preparation system "Multiwave PRO" from Anton Paar at a temperature of 200 °C and a working pressure of 60 bar. The digested solution was diluted to 100 ml and measured using the device mentioned above.

The porosity parameters of samples were derived from the nitrogen physisorption isotherms measured at -196 °C. The measurements were done using a Micromeritics ASAP 2020 device. Before the measurement, the samples were degassed at 400 °C and 4 µbar. The BET (Brunauer-Emmett-Teller) method was used to extract the specific surface area and the BJH (Barrett-Joyner-Halenda) method was employed to calculate the pore size distribution. Additionally, CO_2 adsorption isotherms were measured similarly to the nitrogen isotherms but with an analysis bath temperature of 0 °C instead.

Thermogravimetry measurements of all samples were measured using a Netzsch STA 449 F3 Jupiter device with an TG/DSC probe. To desorb carbon dioxide and water already present in the zeolite micropores, 10-20 mg sample was first heated in an alumina crucible from room temperature (RT) to 500 °C (10 °C min^{-1}) under nitrogen flow and this temperature was

maintained for 2 hours. Afterward, the sample was cooled to RT under nitrogen for 2 hours and subsequently exposed to ambient air for 2 hours. Then, the TG program was run again in nitrogen. To differentiate H_2O and CO_2 during the desorption of ambient species, another set of experiments was performed with the Ca-ZSM-5 sample. First, for desorption 11.4 mg of the sample were heated to 300 °C with a rate of 5 $K \cdot min^{-1}$ and the temperature was held for 10 min under nitrogen. Then the sample was left in the device to cool down to RT (25 °C) still under N_2. Subsequently, another measurement was started with two segments. In the first one the sample was held for 10 min under N_2 and for the second the gas was switched to 400 ppm CO_2 in N_2 (386.9±7.7 ppm CO_2 according to the supplier) and the temperature still held at 25 °C to record the mass gain due to CO_2 adsorption.

1.1.3 Setup and procedure for the direct air capture (DAC):

The setup used for the dynamic adsorption study of CO_2 under flow conditions consists of mass flow controllers supplied from gas cylinders. During the adsorption step the gas fed was synthetic air (0.04 % CO_2 (N4.8), 21 % O_2 (N5.5), 78.96 % N_2 (N5.0)) purchased as a test gas mixture from air-liquid, whereas Ar was fed during the desorption step of the experiment. The MFCs were set to 200 mL/min (20 °C, 1 atm). This gas stream was fed into the inlet of a tubular flow reactor where the adsorbent material could be packed for the adsorption/desorption measurements. The reactor is made of quartz glass and has dimensions of 22 cm length and 2.0 cm inner diameter. The adsorption/desorption of CO_2 / H_2O is monitored continuously online using a mass spectrometer (MS: Pfeiffer Vacuum GSD 350 Omnistar). For data collection, a base pressure of ca. $2 \cdot 10^{-7}$ mbar is applied. Initially, a bypass measurement was performed for 1 to 1.5 h to establish the ion current corresponding to the 400 ppm CO_2 in the synthetic air mixture during which the reactor was purged with Ar. For the measurements of breakthrough curves, after the bypass, the adsorption was performed at 5 °C for 2 h. Then the temperature was increased to 100 °C still under air and after another 60 min, the feed gas was switched to Ar. After reaching a stable MS signal this was taken as a second reference point for 0 ppm CO_2. For the reported CO_2 capacities an error of 20 % was assumed. In the cyclic experiment, water was added to the 100 mL/min synthetic air mixture flow using a syringe pump with a liquid rate of 0.03 mL/h (equivalent to 0.67 mL/min gaseous water (20 °C, 1 atm) and a vaporizer set to 110 °C. Subsequent to the initial bypass the adsorption was performed at 5 °C in the humidified synthetic air mixture. After 30 min the gas was switched to dry argon and the temperature increased to 25 °C for desorption. Before restarting the adsorption by switching to the humidified synthetic air, the reactor was cooled again to 5 °C still under argon.

This cycle of adsorption and desorption was repeated four times in total with a desorption temperature of 150 °C in the last cycle.

1.2. Computational methods:

1.2.1 Monte Carlo (GCMC) simulations

The adsorption of CO_2 in the presence of air components (N_2 and O_2) and water on a modified MFI-type zeolite with a Si/Al ratio of 13, consistent with the structure used in experimental studies, was investigated using grand canonical Monte Carlo (GCMC) simulations and the Metropolis algorithm. The zeolite framework was modeled as rigid, based on previous findings that framework flexibility has only a negligible effect on the adsorption of small molecules like CO_2.[1] Density functional theory (DFT) calculations were employed to enhance the precision and provide deeper insights into the electronic structure of the adsorbates. Adsorbate structures were initially generated and refined using the Dmol^3 module in Materials Studio 2021 (MS). This process ensured accurate geometries and reliable charge distributions. Adsorption isotherms were calculated using the Sorption module in MS, employing the COMPASS III force field to model the interactions between the adsorbates and the zeolite adsorbent. The COMPASS III force field is highly accurate for simulating the structure, dynamics, and thermodynamics of a wide range of materials, including organic, inorganic, and hybrid systems. It offers precise modelling of both intramolecular and intermolecular interactions, making it particularly suitable for studies involving zeolites and gas molecules. Its parametrization enables accurate predictions of physical properties, phase behaviour, and molecular interactions across diverse systems. Long-range electrostatic interactions were handled using the Ewald summation method. This method effectively manages periodic boundary conditions by splitting the electrostatic potential into short-range and long-range components. The short-range interactions were computed directly in real space, while the long-range interactions were calculated in reciprocal space. This approach ensures both accuracy and computational efficiency in evaluating electrostatic contributions, which are crucial in systems where periodicity and electrostatic forces significantly influence adsorption behaviour. For chemical equilibration, particle exchange with the reservoir was crucial. In our simulations, a total of $1{\cdot}10^5$ Monte Carlo cycles were conducted, with the first $1{\cdot}10^4$ cycles used as initialization or equilibration cycles to allow the system to reach a steady state before data collection. Periodic boundary conditions were applied to the simulation box to eliminate edge effects and mimic an infinite system. This ensured that particles near the boundaries experienced the same environment

as those within the interior, maintaining consistency in particle interactions and overall system behaviour throughout the simulation.

1.2.2. Molecular modelling and computational details

The binding and adsorption behaviour of CO_2 on a modified MFI-type zeolite with a Si/Al ratio of 13 was investigated at a molecular level using force-field-based molecular dynamics (MD) simulations. The MFI surface was modeled by replicating the MFI unit cell (lattice constants: a = 20.022, b = 19.899, and c = 13.383 Å) twice along the a-direction, twice along the b-direction, and three times along the c-direction. This replication resulted in a supercell with dimensions x = 40.044, y = 39.798, and z = 40.149 Å, comprising 3456 atoms (1068 Si, 84 Al, and 2304 O). Three MFI surface planes ([100], [010], and [001]) were modeled, with 60 CO_2 molecules placed in a 100 Å vacuum region perpendicular to each surface plane, as shown in Figure S7. All simulation boxes were neutralized by incorporating 42 sodium ions (Na^+). After relaxation and during production, these ions localized near Al–O environments (see Fig. S15). The Al site positions used in the models are shown in Figure S7 and Figure S15 and were initially assigned from the MC simulations. The CO_2-MFI system was modeled using the compatible CLAYFF force field for the MFI surface Na ions,[2] and the CHARMM force field for CO_2.[3] The simulations were carried out under periodic boundary conditions in all three spatial dimensions. The nonbonded parameter set (charge q, Lennard–Jones σ (nm), ε (kJ mol^{-1})) used in the GROMACS topologies for and MFI and CO_2 are: Si_{MFI} (2.100, 0.3302, 0.000007701), Al_{MFI} (1.600, 0.3302, 0.000007701), O_{MFI} (-1.050, 0.316554146154, 0.6501936), Na (1.000, 0.235, 0.5443), C_{CO2} (0.686, 0.278494939291, 0.2426720), and O_{CO2} (-0.343, 0.301480126219, 0.6903600).

For each molecular model, energy minimization was performed, followed by an equilibration step and a final production run of 500 ns using canonical ensemble (NVT, i.e., constant number of atoms N, volume V, and temperature T) MD simulations. The MD simulations were conducted with a time step of 2 fs, employing a 12 Å straight cutoff and the Verlet neighbor list scheme. Short-range cutoffs were rcoulomb = 1.2 nm and rvdw = 1.2 nm with a force-switch between 1.0–1.2 nm for vdW. Long-range electrostatics employed PME. Bonds involving hydrogen were constrained with LINCS. Temperature control was maintained at 27 °C using the velocity-rescaling thermostat with a coupling constant of 0.1 ps.[4] The interaction energy between CO_2 and the MFI framework was calculated throughout the production trajectory, considering electrostatic Coulomb and van der Waals interactions. All simulations and analyses were performed using the GROMACS software package, version 2019.4.[5, 6]

To investigate why CO_2 preferentially adsorbs in the presence of moisture under ambient conditions at the molecular level, we complemented our MD simulations of pure CO_2 with six additional simulations on two systems: (i) 60 H_2O molecules and (ii) a 1:1 mixture of 30 CO_2 and 30 H_2O molecules. Each system was initially adsorbed onto the three MFI zeolite surface planes ([100], [001], and [010]) to assess the influence of surface orientation. These simulations reveal how surface structure, electrostatics, and dispersion interactions collectively govern CO_2 adsorption, H_2O–CO_2 co-adsorption, and directional transport within the zeolite framework.

We note that the simulation model, particularly the 1:1 CO_2/H_2O mixture (30 molecules each), does not replicate the exact gas-phase composition used in the experiments, which involved a continuous flow of synthetic air containing 0.04% CO_2, with or without H_2O vapour at 80% relative humidity. Instead, this simplified setup was chosen to enable molecular-level exploration of competitive adsorption and diffusion under computationally tractable conditions. Although the modeled concentrations of CO_2 and H_2O are higher than those in the experimental system, the observed trends, such as the stronger surface affinity and reduced mobility of H_2O relative to CO_2, are qualitatively consistent with experimental findings. Accordingly, these simulations are intended to offer mechanistic insights rather than reproduce the exact partial pressures or flow dynamics of the experimental conditions.

2. Supporting Tables:

Table S1: Summary of textural properties and porosity of ion-exchanged M-ZSM-5 samples and the pristine material with a nominal molar ratio of Si/Al = 13.6.

Parameter	ZSM-5	Zn-ZSM-5	Mg-ZSM-5	Ca-ZSM-5
metal loading (weight %)	-	0.8	0.30	0.45
metal loading ($mmol{\cdot}g^{-1}$)	-	0.122	0.123	0.112
Metal/Al ratio (mol-%)		18.7	7.6	12.4
SSA_{BET} / $m^2\ g^{-1}$	407	390	403	407
V_{BJH} / $cm^3\ g^{-1}$	0.164	0.121	0.123	0.125
Pore diameter/ nm	0.84	0.84	0.84	0.88

Table S2: The amount of adsorbed CO_2 on different ZSM-5-based samples during the dynamic adsorption-desorption experiments.

Adsorbent	Desorbed CO_2 / $mmol{\cdot}g^{-1}$
ZSM-5	0.031
Ca-ZSM-5	0.028
Mg-ZSM-5	0.024
Zn-ZSM-5	0.013

3. Supporting Figures:

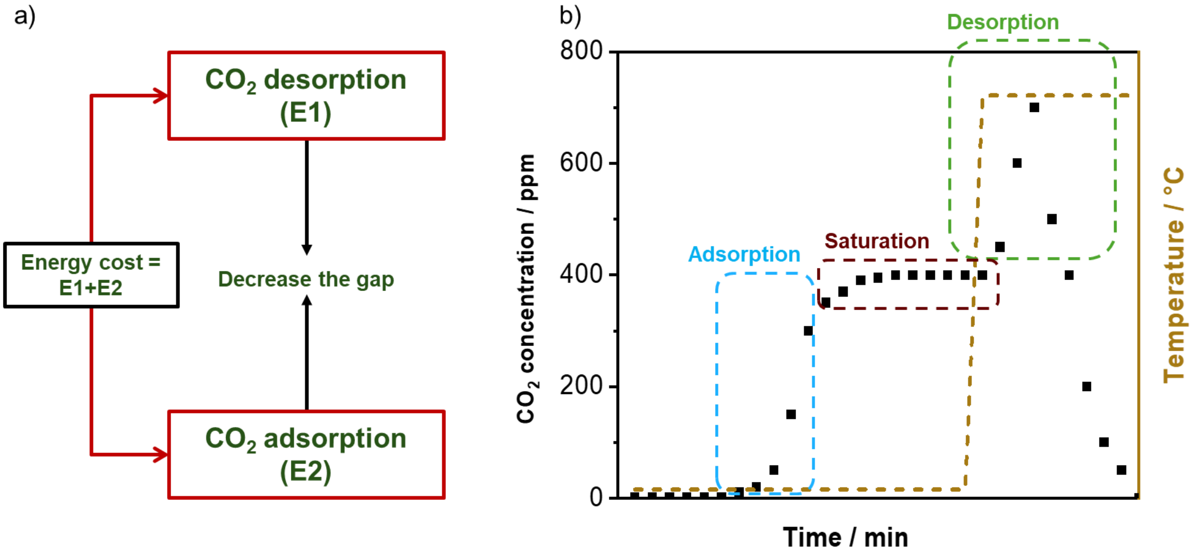

Figure S1: (a) Illustration of the energy demand of the adsorption/desorption window in the CO_2-DAC process. (b) illustration of the breakthrough curve during the adsorption/desorption process in the capture of CO_2 under flow conditions.

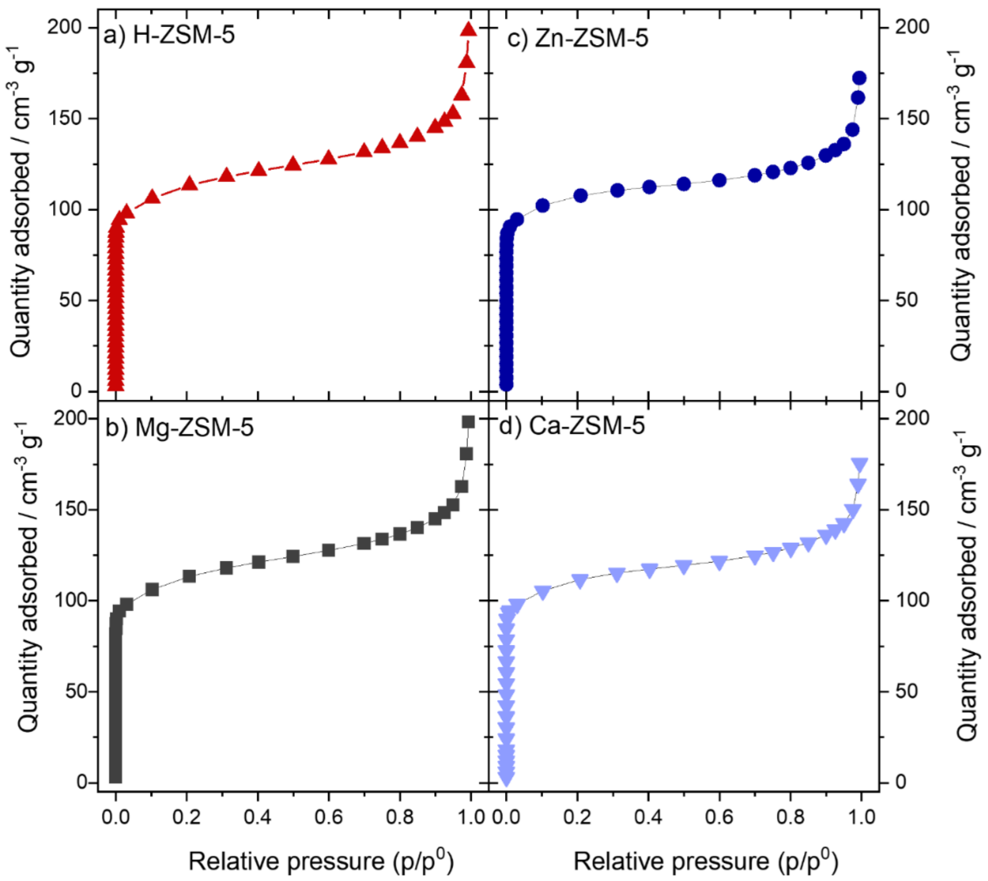

Figure S2: N_2 adsorption/desorption isotherms of the as-prepared frameworks (a: H-ZSM-5; b: Mg-ZSM-5; c: Zn-ZSM-5; d: Ca-ZSM-5). Before each adsorption experiment, the sample was degassed for 2 h at 200 °C and 4 µbar.

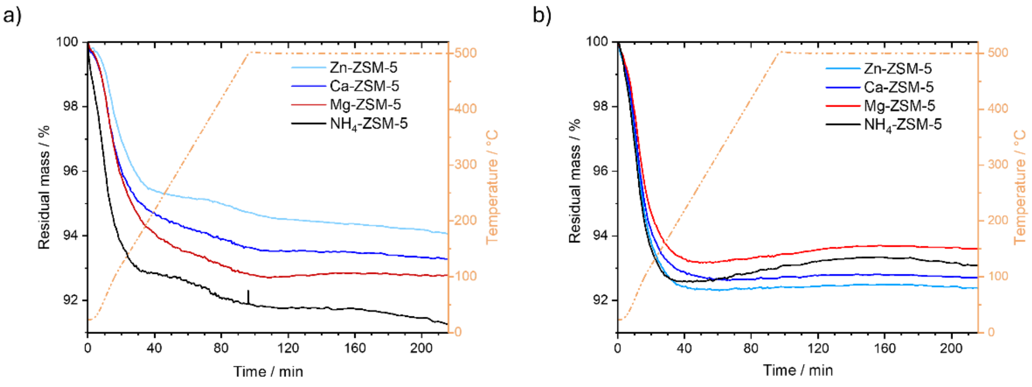

Figure S3: (a) Thermogravimetric analysis (TGA; Left Y-axis: residual mass; right Y-axis: temperature; X-axis: time) of the as-prepared ZSM-5 adsorbents in N_2. (b) TGA profile of adsorbents after the first TGA profile (shown in part a) followed by cooling to RT in N_2 for 2 hours and subsequent exposure to ambient air for 2 h.

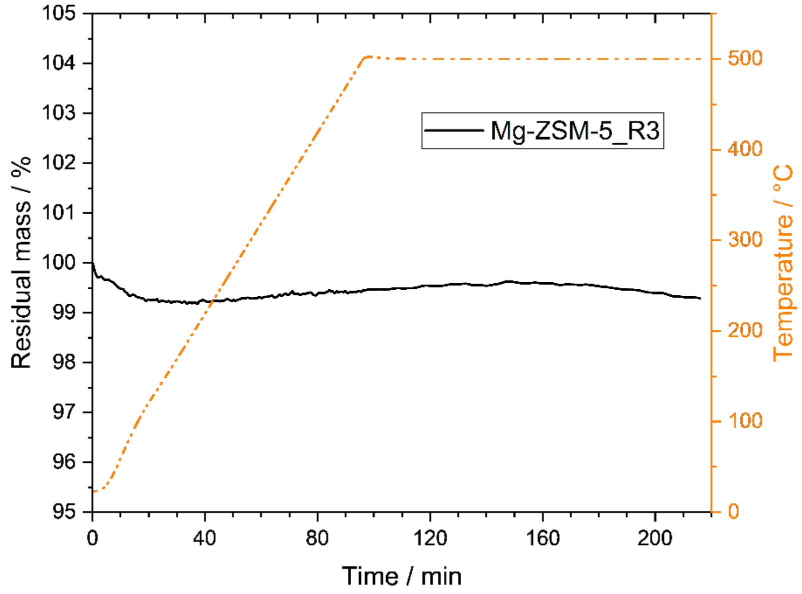

Figure S4: Thermogravimetric analysis (TGA) profile collected on the Mg-ZSM-5 adsorbent after a preceding TGA step followed by keeping the adsorbent under a flow of N_2 for 16 h at RT.

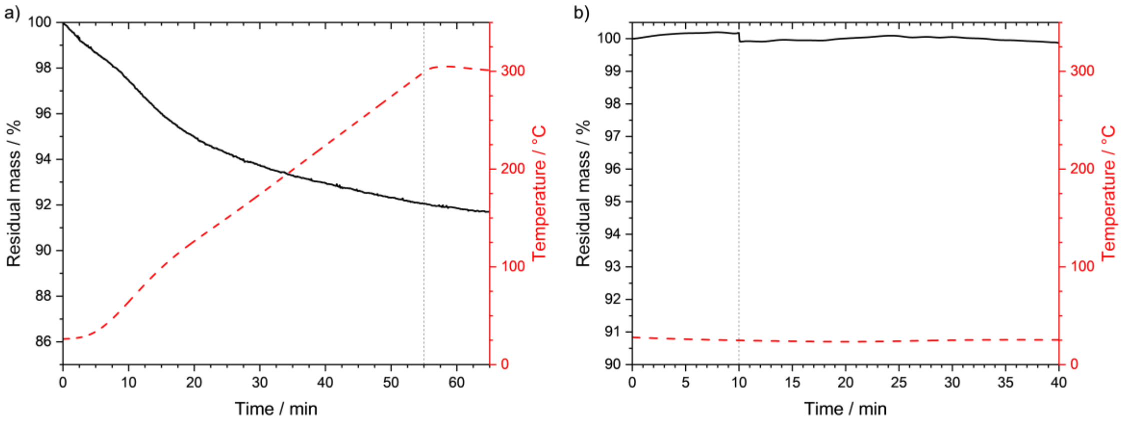

Figure S5: TG curves of Ca-ZSM-5 under a) pure nitrogen to desorb species from ambient air and b) N_2 (segment 1) and 400 ppm CO_2 in N_2 (segment 2). Dotted vertical lines indicate segment transition. Between the measurements the sample was cooled to RT under N_2 in the device.

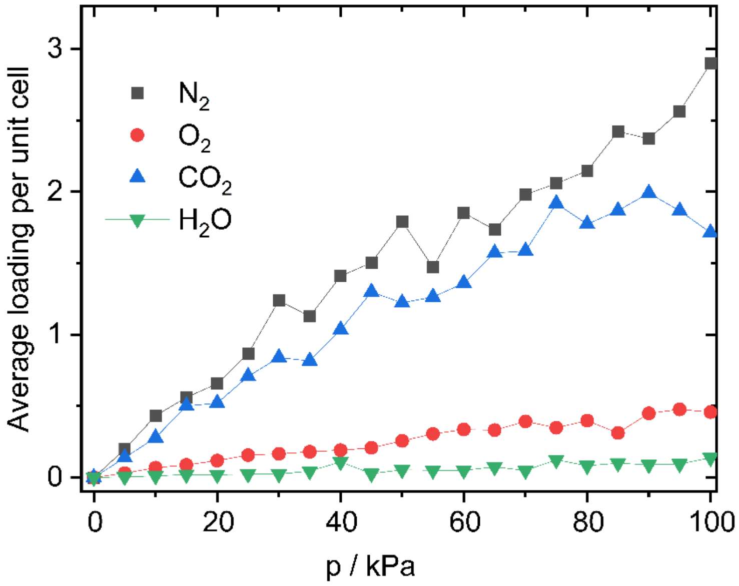

Figure S6: Simulated adsorption isotherms of the gas mixture (72.48 % N_2, 13.52 % O_2, 4 % CO_2, and 10 % H_2O) in the modified MFI structure at 25 °C.

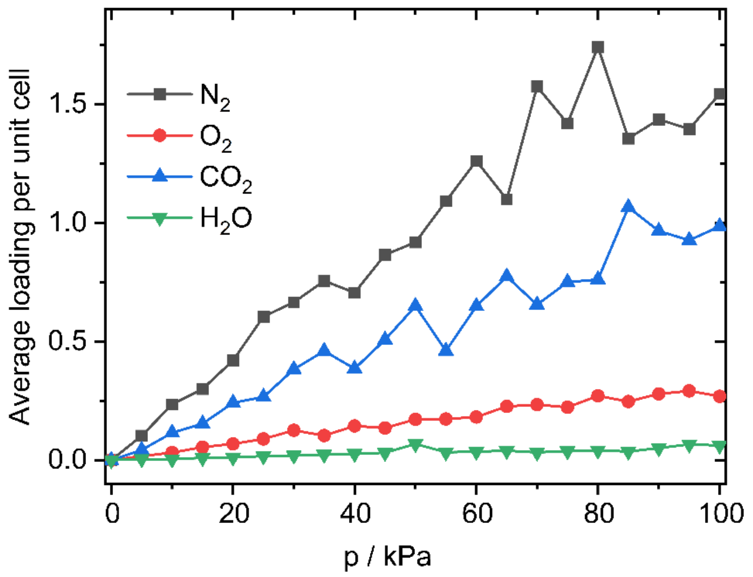

Figure S7: Simulated adsorption isotherms of the gas mixture (72.48 % N_2, 13.52 % O_2, 4 % CO_2, and 10 % H_2O) in the modified MFI structure at 50 °C.

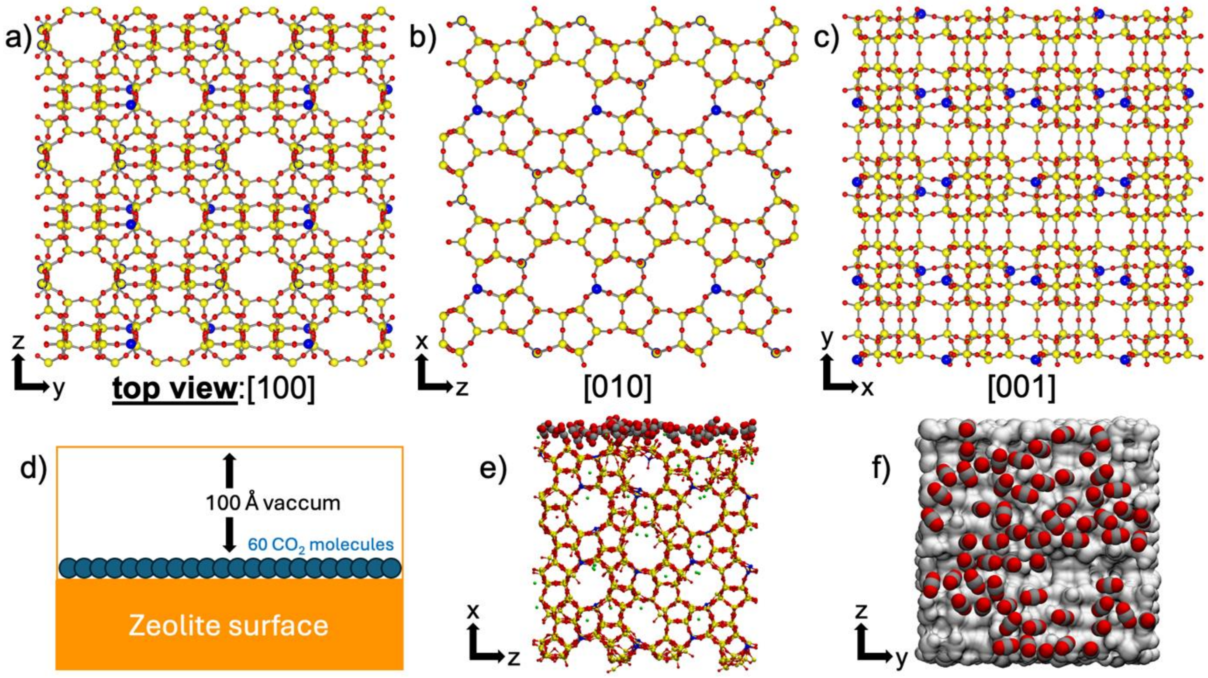

Figure S8: (a–c) Top views of the three modeled MFI surface planes, (d) schematic representation of the molecular modeling approach for CO_2 adsorption on the MFI surface, and (e) side view and (f) top view of the initial configuration with 60 CO_2 molecules placed on the [100] MFI surface plane. Atoms are colour-coded as follows: Si (yellow), Al (blue), Na (green), C (gray), and O (red). The MFI surface in (f) is presented in light gray.

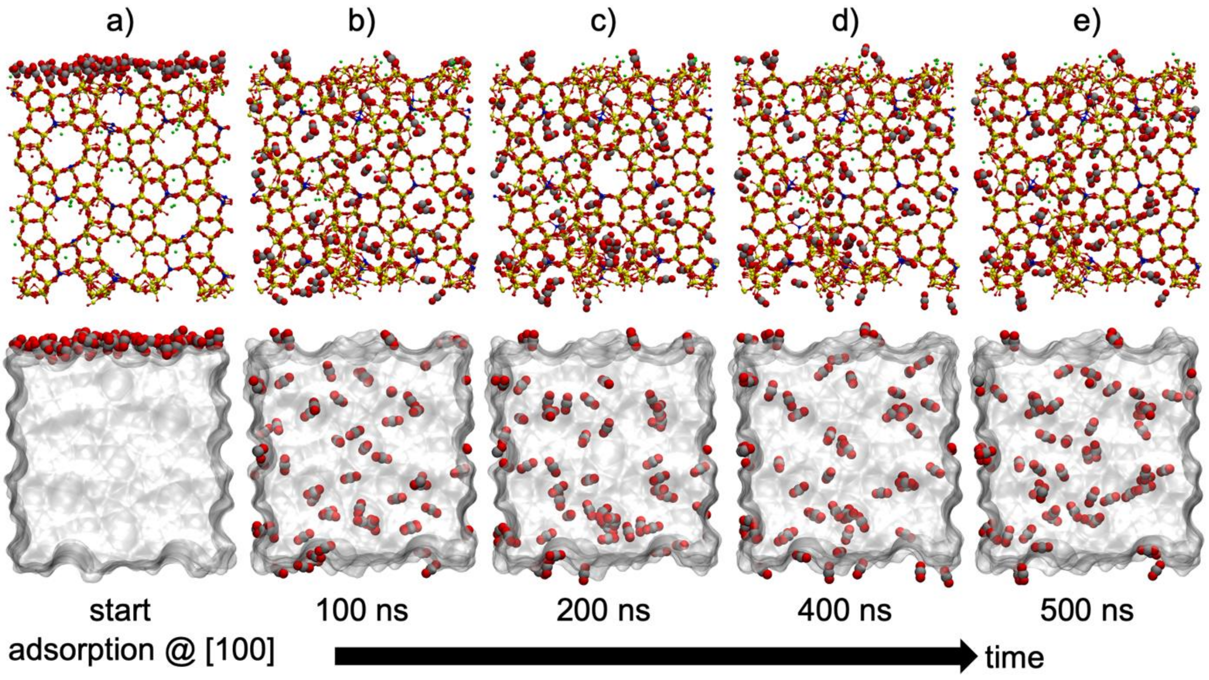

Figure S9: Side-view snapshots captured during the MD simulation of CO_2 adsorption on the [100] MFI surface plane. Atoms are depicted in distinct colours: yellow for Si, blue for Al, green for Na, gray for C, and red for O. In the bottom representations, the MFI surfaces are shown as a transparent white surface to enhance visualization of the diffused CO_2 through the MFI structure.

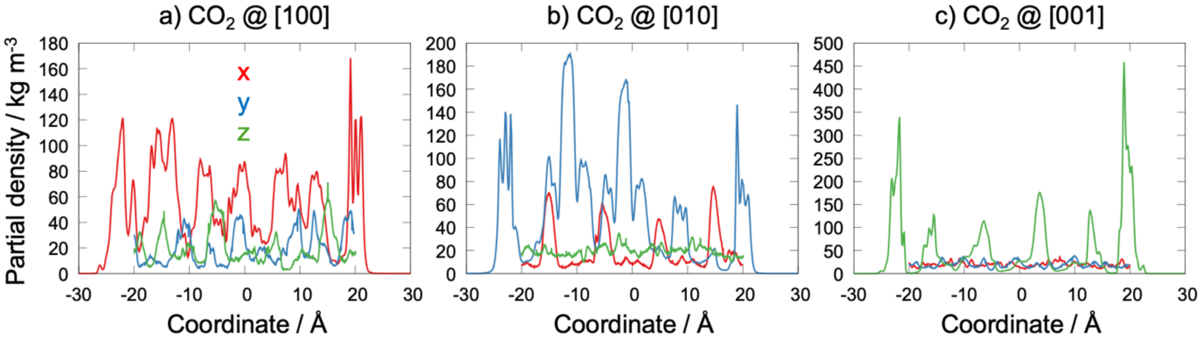

Figure S10: The partial density of CO_2 molecules along each dimension (x, y, and z) for the three MFI surface planes. The center of the MFI surface was taken as zero along each respective surface plane.

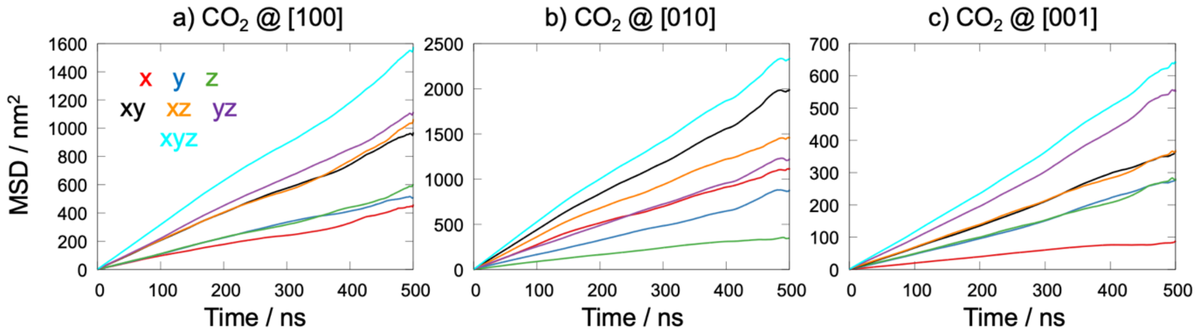

Figure S11: The overall mean square displacement (MSD) in the xyz directions, along with its individual components in each single dimension (x, y, and z), and for plane (xy, xz, and yz) of CO_2 for the three MFI surface planes.

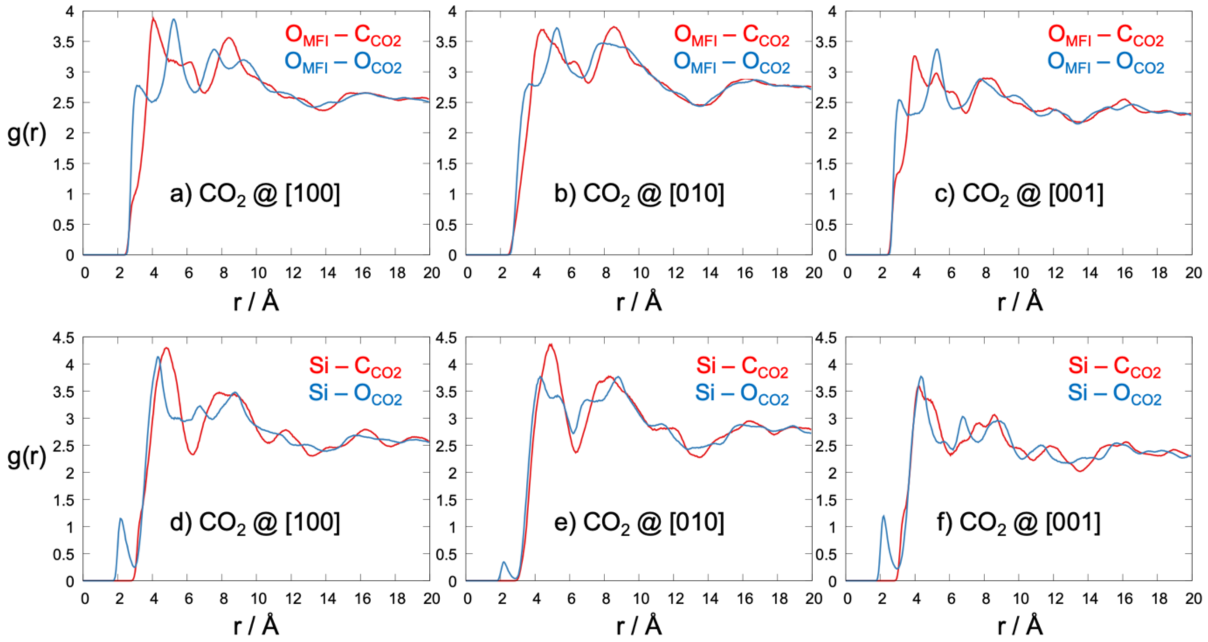

Figure S12: The radial distribution function, g(r), of the distance between both C and O atoms of CO_2 and the MFI-O atoms (top three panels) and Si atoms (bottom three panels) for the three MFI surface planes.

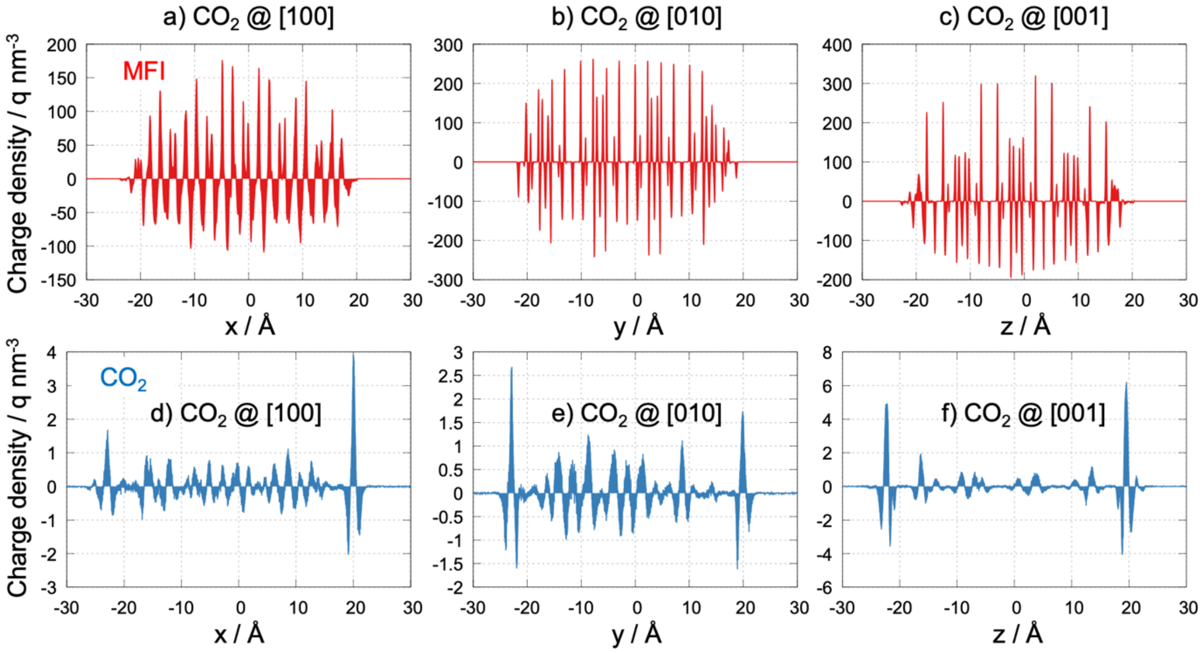

Figure S13: The charge density of the MFI (top three panels) and CO_2 (bottom three panels) along the axis perpendicular to the MFI surface plane is given in the corresponding panel. The center of the MFI surface was taken as zero along each respective axis.

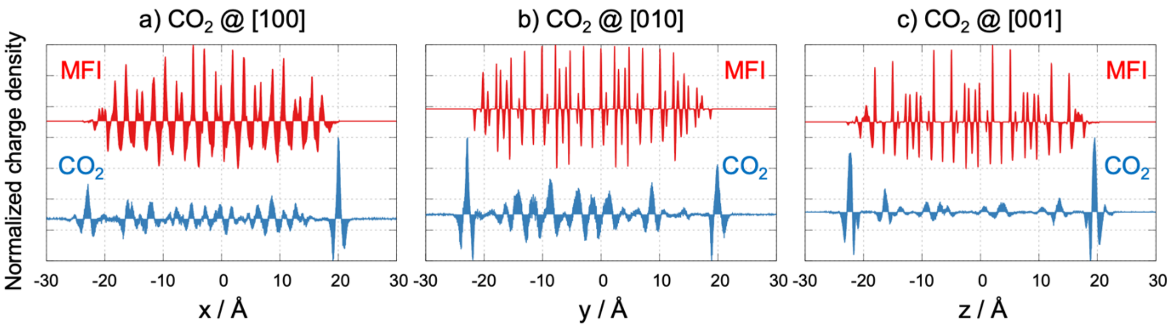

Figure S14: The normalized charge density of both MFI and CO_2, as presented in Figure S12, along the axes perpendicular to the MFI surface planes given in the respective panel.

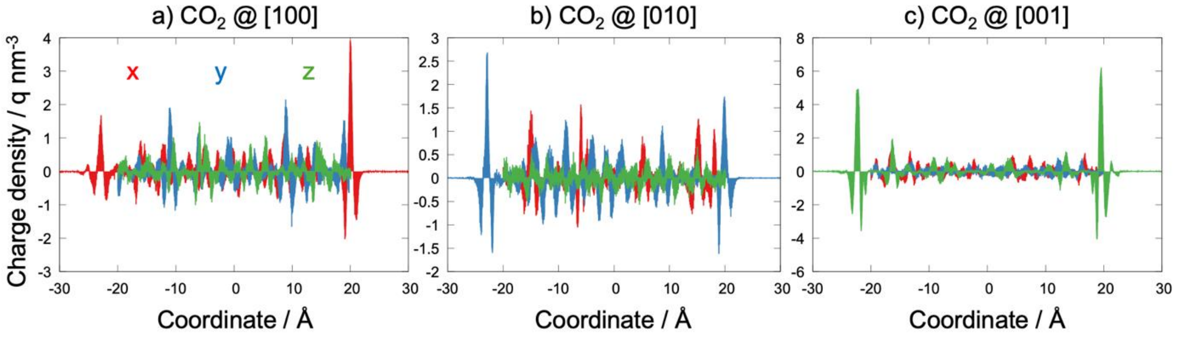

Figure S15: The charge density of CO_2 along the x-, y-, and z-axes for the three MFI surface planes. The center of the MFI surface was taken as zero along each respective axis.

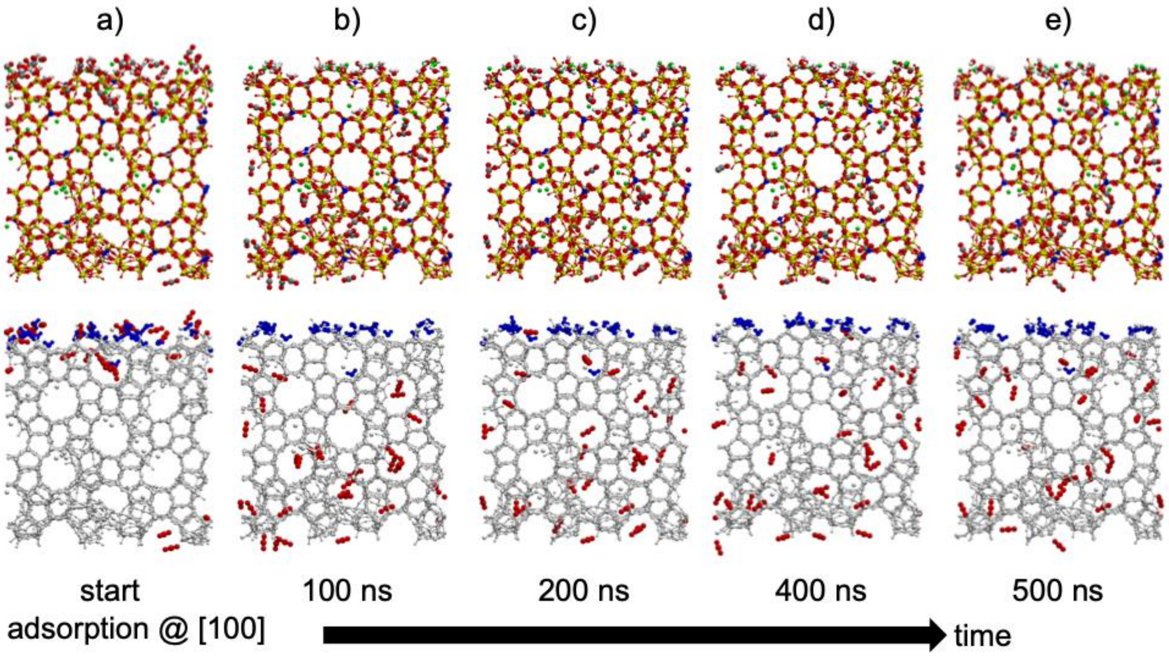

Figure S16: Side-view snapshots from an MD trajectory showing co-adsorption of 30 CO_2 and 30 H_2O on the MFI [100] surface over time. Two visualization schemes help track uptake: Top, atoms coloured by element: Si (yellow), Al (blue), Na (green), C (gray), O (red), H (white). Bottom, the MFI framework (including Na^+) is rendered in white to highlight guest transport; CO_2 is shown in red and H_2O in blue, emphasizing CO_2 diffusion through the MFI channels and surface-trapped H_2O.

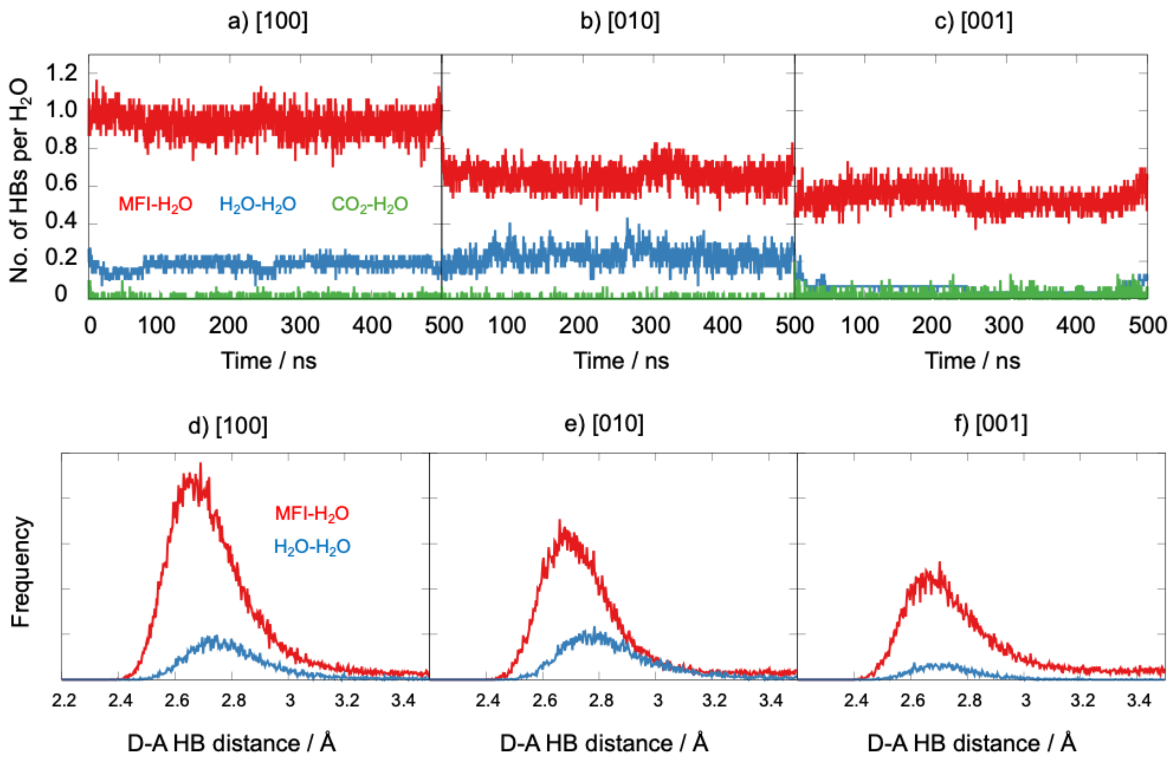

Figure S17: Hydrogen bonding in CO_2/H_2O mixtures on MFI surfaces. (a–c) Number of hydrogen bonds per H_2O molecule between MFI and water (MFI–H_2O), within water molecules (H_2O–H_2O), and between CO_2 and water (CO_2–H_2O) for the 30 CO_2 + 30 H_2O system on the [100] (a), [010] (b), and [001] (c) surfaces. (d–f) Distributions of donor–acceptor (D–A) hydrogen-bond (HB) distances for MFI–H_2O and H_2O–H_2O on [100] (d), [010] (e), and [001] (f), respectively.

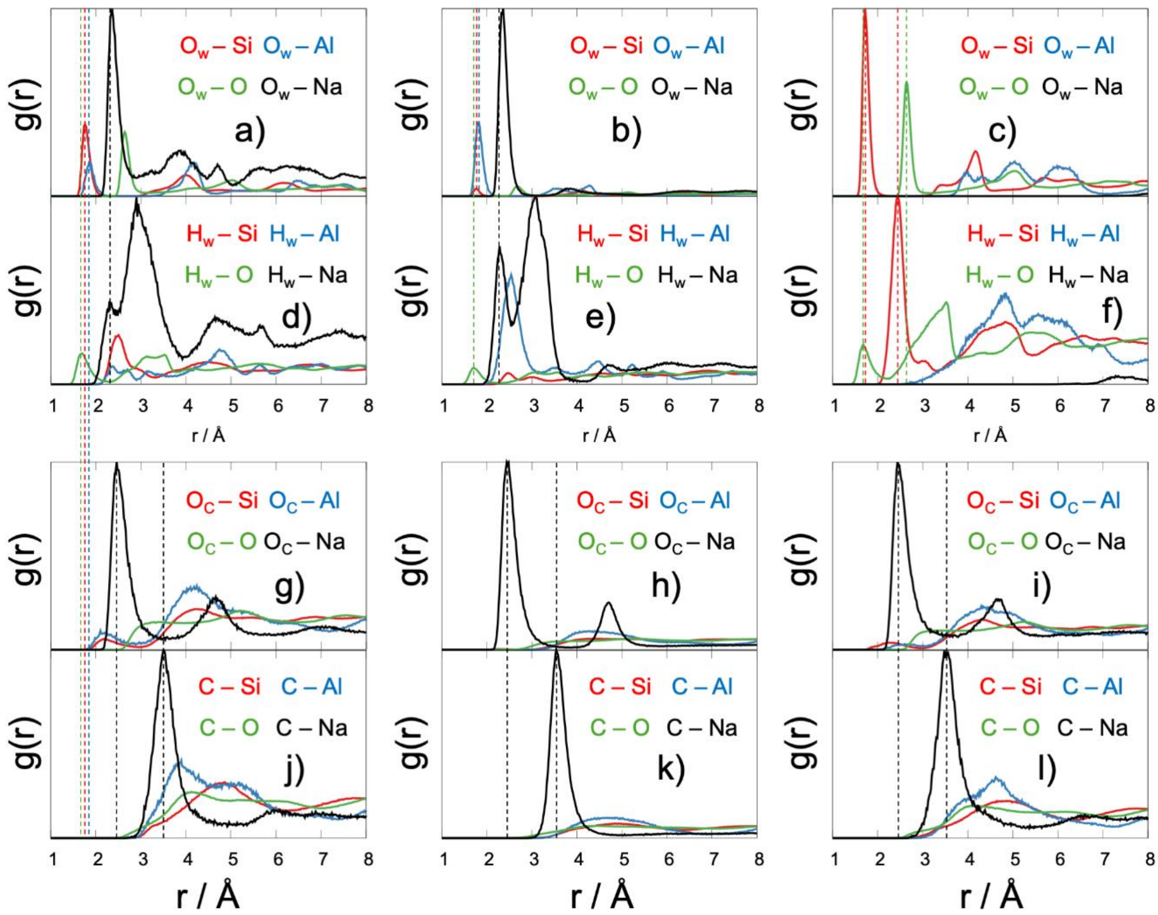

Figure S18. Radial distribution functions, $g(r)$, in ZSM-5 (MFI) for adsorbates relative to Na^+ and framework atoms (Si, Al, O), resolved by crystallographic direction to highlight hannel/intersection anisotropy: (a–c) water oxygen (Ow) along [100], [010], [001]; (d–f) water hydrogen (Hw); (g–i) CO_2 oxygen (OC); and (j–l) CO_2 carbon (C).

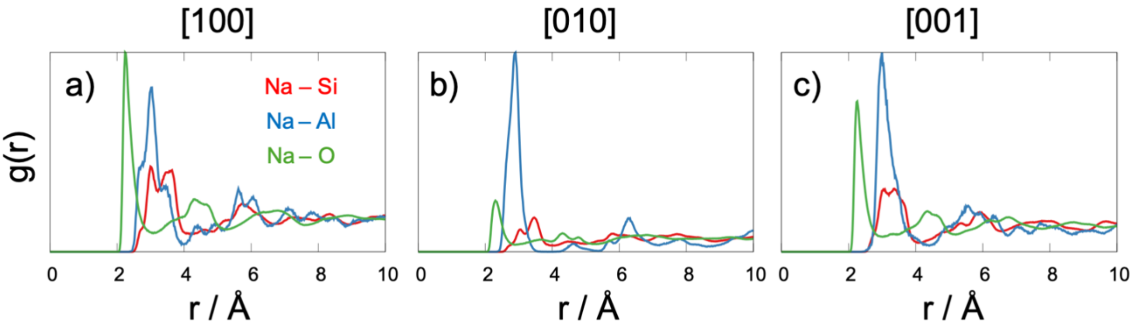

Figure S19. Radial distribution functions, $g(r)$, for Na^+–framework pairs (Na–Si, Na–Al, Na–O) in ZSM-5 (MFI), resolved by crystallographic direction: (a) [100], (b) [010], (c) [001].

Additional Informaiton related to calculations in Figure S18 /S19:

<u>Site occupancy</u>: To quantify site occupancies, we analyze 500-ps atomistic MD trajectories of a 30 CO_2 + 30 H_2O mixture on the [100], [010], and [001] surfaces of Na–MFI, determining how CO_2 and H_2O partition among surface/micropore environments and how long they persist at each site. At each saved frame, every molecule is assigned a primary site by instantaneous geometry: **Na^+** if any guest O lies within 3.0 Å of Na^+; **O_{HB}** (water only) if an H··· O_{MFI} (O_{MFI} = framework O atoms) contact satisfies H··· O_{MFI} ≤ 2.3 Å and ∠(O–H···O_{MFI}) ≥ 150° (strict H-bond); otherwise **O_{vdW}** if any guest heavy atom is within 3.5 Å of O_{MFI}; frames failing all criteria are **FREE**. The occupancy of site ***s*** is the fraction of simulation time a molecule is labeled at ***s***, averaged over all molecules of a given species. Here we also quantify a conditional co-localization metric for CO_2 given the local water environment.

<u>Residence time calculaitons</u>: Residence times are computed by converting per-frame site labels into continuous episodes and stitching brief interruptions (rapid recrossing) shorter than a fixed tolerance (default 0.5–2.0 ns), so high-frequency flicker does not artificially split a single visit. Episode durations converted using the saved frame spacing and any stride, define empirical survival functions $S(t)$. The mean residence time (MRT) for a site is the arithmetic mean of episode lengths (equivalently, the area under $S(t)$). We report per-site MRTs and medians, and 95% confidence intervals for occupancies. Sensitivity analyses with respect to the stitching tolerance and the H-bond distance cutoff (2.3–2.6 Å) do not change the qualitative conclusions; we therefore use the strict 2.3 Å criterion in the main analysis.

Across planes, both occupancy and kinetics show strong anisotropy consistent with "reduced competition under our conditions." On [100], H_2O partitions mainly between **O_{HB}** (0.47) and **O_{vdW}** (0.38) with a minor **Na^+** fraction (0.15), while CO_2 is predominantly **O_{vdW}** (0.76) with a smaller **Na^+** component (0.24). H_2O episodes at [100] are comparatively long (MRT ≈ 128–131 ns at **O_{HB}**/**O_{vdW}**), whereas CO_2 visits are shorter (**O_{vdW}** 11.8 ns, **Na^+** 3.3 ns), reflecting frequent hopping. On [010], H_2O shows a large **Na^+** occupancy (0.42) and smaller **O_{HB}**/**O_{vdW}** (≈0.26/0.28), while CO_2 flips: **Na^+** dominates (0.75) and **O_{vdW}** is 0.24; both species exhibit short lifetimes (H_2O MRTs mostly 7–15 ns, CO_2 (**O_{vdW}**) 2.7 ns, CO_2 (**Na^+**) 14.9 ns), consistent with faster exchange. On [001] plane, H_2O exhibits no Na^+ binding and instead favors **O_{vdW}** (0.65) over **O_{HB}** (0.35), with MRTs of 86.4 ns (**O_{vdW}**) and 51.9 ns (**O_{HB}**). CO_2

partitions between **O$_{vdW}$** (0.68) and **Na$^+$** (0.31) with short lifetimes, 9.2 ps and 3.5 ns, respectively, indicating that, even in the presence of surface-bound water, CO_2 rapidly accesses and transits the micropores.

Conditionals reinforce this picture: when nearby H_2O is **O$_{HB}$** or **O$_{vdW}$**, CO_2 is overwhelmingly **O$_{vdW}$** (e.g., 0.67–0.96 across planes), indicating that water adjacent to the framework biases CO_2 toward wall/physisorption rather than cation binding. Elevated CO_2@**Na$^+$** probabilities appear only when water itself is **Na$^+$**-bound (e.g., 0.86 on [100], 0.78 on [010]). Taken together, H_2O predominantly occupies surface H-bond/vdW states (or **Na$^+$** on [010]) with lifetimes comparable to or exceeding those of CO_2, yet CO_2 still retains substantial, and often rapid, access to **O$_{vdW}$** (and to **Na$^+$** where available), consistent with reduced direct competition and sustained CO_2 infiltration into the channels under our conditions.

References:

1. Wotzka, A.; Jorabchi, M. N.; Wohlrab, S., *Membranes* **2021,** *11* (6), 439. DOI 10.3390/membranes11060439.
2. Cygan, R. T.; Liang, J.-J.; Kalinichev, A. G., *J. Phys. Chem. B* **2004,** *108* (4), 1255-1266. DOI 10.1021/jp0363287.
3. Jo, S.; Kim, T.; Iyer, V. G.; Im, W., *J. Comput. Chem.* **2008,** *29* (11), 1859-1865. DOI doi.org/10.1002/jcc.20945.
4. Bussi, G.; Donadio, D.; Parrinello, M., *J. Chem. Phys.* **2007,** *126* (1). DOI 10.1063/1.2408420.
5. Abraham, M. J.; Murtola, T.; Schulz, R.; Páll, S.; Smith, J. C.; Hess, B.; Lindahl, E., *SoftwareX* **2015,** *1*, 19-25. DOI 10.1016/j.softx.2015.06.001.
6. Van Der Spoel, D.; Lindahl, E.; Hess, B.; Groenhof, G.; Mark, A. E.; Berendsen, H. J., *J. Comput. Chem.* **2005,** *26* (16), 1701-1718. DOI 10.1002/jcc.20291
